# Diabetes Moderates the Link between Personality Traits and Self-Rated Health (SRH)

**DOI:** 10.3390/healthcare11152149

**Published:** 2023-07-28

**Authors:** Weixi Kang

**Affiliations:** Department of Brain Sciences, Imperial College London, London W12 0BZ, UK; wk20@imperial.ac.uk

**Keywords:** diabetes, personality, big five, self-rated health

## Abstract

Objectives: The primary objective of this study is to explore the relationship between personality traits and self-rated health (SRH) in individuals with diabetes, while also comparing these associations with a group of healthy controls. Methods: The data for this study were obtained from the UK Household Longitudinal Study (UKHLS), comprising a sample of 1860 diabetes patients and 12,915 healthy controls who were matched in terms of age and sex. Hierarchical linear regression was utilized to analyze the data. The analysis included demographic variables such as age, sex, monthly income, highest educational qualification, marital status, and psychological distress assessed through the GHQ-12, personality traits, including Neuroticism, Agreeableness, Openness, Conscientiousness, and Extraversion, and diabetes status (0 for diabetes patients, 1 for healthy controls) as predictors. Interactions between personality traits and diabetes status were also included as predictors, with SRH serving as the dependent variable. Additionally, separate multiple regression analyses were conducted for diabetes patients and healthy controls, incorporating demographic variables, psychological distress, and personality traits as predictors, while SRH remained the dependent variable. Results: The findings of this study indicate that diabetes significantly moderates the association between Neuroticism and SRH. Specifically, both Neuroticism and Extraversion were negatively associated with SRH, whereas Openness and Conscientiousness exhibited a positive association with SRH in healthy controls. However, among diabetes patients, only Conscientiousness showed a positive association with SRH. Conclusion: Personality traits predict SRH in people with and without diabetes differently. Healthcare professionals and clinicians should try to come up with ways that improve SRH and thus better outcomes in diabetes patients based on the findings from the current study.

## 1. Introduction

Self-rated health (SRH) is an individual’s subjective assessment of their own health and has been widely utilized as a measure of overall health in population-based health research [1]. Despite its non-specific nature, SRH has demonstrated good predictive validity, as evidenced by its associations with various health conditions such as stroke, lung disease, arthritis, functional impairment, cardiovascular disease, depression, and overall mortality [2,3]. In the context of diabetes, SRH has been linked to numerous risk factors, including smoking, alcohol consumption, physical inactivity, unhealthy dietary choices, obesity, and genetic factors, all of which may contribute to the development of diabetes [4,5,6]. Previous studies have also observed a negative correlation between SRH and inflammatory markers, which are known risk factors for diabetes [7,8,9]. Multiple cross-sectional studies have reported a negative association between SRH and diabetes [10,11], and two cohort-based studies have found that SRH is inversely related to the risk of developing diabetes over time [12,13].

There are some debates in the literature regarding what has really been incorporated in the SRH, either consciously or subconsciously. Aside from demographics and medical information, accumulating evidence seems to suggest that SRH may reflect people’s representation at two levels, including conscious awareness of health complaints (e.g., weakness, fatigue, and tiredness), which reflects the unmeasured severity of symptoms that individuals are aware of [14,15,16], and the perception of interoceptive processes and somatic reactions (e.g., heartbeat), which are perceived by sensation but related to aforementioned health complaints that will be integrated to rate one’s health status [3,17,18].

Furthermore, prior research has demonstrated the significance of individuals’ perception of their illness and self-assessment of health in relation to various aspects of disease recovery, as supported by theoretical models. One such model is the Common Sense Model (CSM) proposed by Leventhal et al. [19,20], which suggests that when evaluating their self-perception of health, individuals consider factors such as experienced symptoms attributed to the illness (identity), beliefs regarding the causes of the illness (cause), impact on quality of life and functioning (consequences), beliefs about the duration and cyclical nature of the illness (timeline), perceived controllability through personal coping behaviors and medical treatment (control), presence of negative emotions related to the illness (emotional representations), and overall understanding of the condition (coherence) [21]. These theories indicate that personal, environmental, and cultural factors contribute to SRH and may also influence coping strategies. Therefore, individual factors, such as personality traits, play a crucial role in predicting SRH. Numerous studies have examined the associations between personality traits and SRH, with one recent study analyzing eight large cohorts and finding a negative association between Neuroticism and SRH, while Extraversion, Openness, Agreeableness, and Conscientiousness showed positive associations [22]. In the context of diabetes, SRH has been linked to physical functioning, depression, diabetes-related complications, and lifestyle behaviors.

Despite some previous studies examining the connections between personality traits and SRH in individuals without chronic illnesses, there remains a scarcity of research specifically investigating how personality traits can serve as predictors of SRH among patients with diabetes. This knowledge gap is particularly significant considering that SRH is intricately linked to various health outcomes. Therefore, the primary aim of this study is to comprehensively explore the intricate relationship between personality traits and SRH in individuals diagnosed with diabetes, while concurrently comparing these associations with a control group of healthy individuals. Investigating the impact of personality traits on SRH among diabetes patients holds substantial implications for improving our understanding of the psychological factors that influence health outcomes in this specific population. By identifying which personality traits are associated with higher or lower SRH ratings, we can gain valuable insights into the potential psychological mechanisms underlying subjective health perceptions among individuals managing diabetes. Furthermore, comparing these associations with a control group of healthy individuals will provide valuable comparative data, enabling a better assessment of the unique role of personality traits in shaping SRH specifically within the context of diabetes.

Ultimately, the findings from this study will contribute to the existing literature on SRH and personality traits by providing novel insights into the unique dynamics between these factors within the diabetes population. Such knowledge can inform the development of targeted interventions aimed at promoting better SRH and overall well-being among individuals living with diabetes. Moreover, the comparative analysis with healthy controls will allow for a better understanding of the distinctive role that personality traits play in influencing SRH in the context of chronic illness versus overall health, shedding light on potential differences or similarities in these relationships.

In summary, this research endeavor seeks to bridge the gap in knowledge regarding the interplay between personality traits and SRH specifically in diabetes patients. By investigating these associations and comparing them with a healthy control group, this study aims to generate insights that have practical implications for healthcare professionals, psychologists, and researchers alike, facilitating the development of targeted interventions and strategies to enhance SRH and overall quality of life for individuals managing diabetes.

## 2. Methods

### 2.1. Data

The current study extracted data from Understanding Society: the UK Household Longitudinal Study (UKHLS), which has been collecting annual information from the original sample of UK households since 1991 [23]. The initial step involved participants responding to a question regarding their clinical diagnosis of diabetes during the first wave of data collection, which took place between 2009 and 2010. Subsequently, participants were asked again in each subsequent wave if they had received a new diagnosis of diabetes. Additionally, during the third wave (collected between 2011 and 2012), participants completed questionnaires pertaining to personality traits, demographic information, and psychological distress. To establish a comparison group, controls matching the participants in terms of age and sex were chosen from individuals who had not received a clinical diagnosis of diabetes. Participants with any missing data were excluded from further analysis. Consequently, the final sample consisted of 1860 individuals with diabetes and 12,915 healthy controls who were matched in terms of age and sex. Descriptive statistics can be found in Table 1.

### 2.2. Measures

#### 2.2.1. Diabetes

Participants were asked to indicate whether a healthcare professional had ever diagnosed them with diabetes by responding to the question, “Has a doctor or other health professional ever told you that you have any of these conditions? Diabetes”. Self-reported diabetes has been recognized as a reliable indicator of diabetes status across different countries [24,25,26,27,28,29,30].

#### 2.2.2. Personality Traits

The personality traits of the participants were assessed using the 15-item edition of the Big Five Inventory, employing a Likert scale that ranged from 1 (“strongly disagree”) to 5 (“strongly agree”). Whenever necessary, the scores were reversed. The specific questions utilized to inquire about participants’ traits can be accessed at the following link: https://www.understandingsociety.ac.uk/documentation/mainstage/dataset-documentation/term/personality-traits?search_api_views_fulltext=, accessed on 11 November 2022. This 15-item version of the Big Five appears to have good validity and reliability [31,32].

#### 2.2.3. SRH

The reliability of this single subjective health measurement is considered moderate, as evidenced by previous research [33]. To make it more intuitive, the SRH scores were reverse coded, meaning that now a score of 1 represents “very poor” and a score of 5 represents “excellent”.

#### 2.2.4. Demographics Controls

The participants provided information regarding their demographic variables, which encompassed age, sex, income (monthly), education, marital status, and psychological distress assessed using the GHQ-12 [34]. More specifically, age, income (monthly), and psychological distress were coded according to their original values, while sex was coded as either male or female, and education was coded as below college or college-level.

#### 2.2.5. Analysis

In order to understand if diabetes moderates the associations between personality traits and SRH, a hierarchical linear regression approach was employed to examine the data. The analysis considered demographic variables, such as age, sex, income (monthly), education, marital status, and psychological distress measured by the GHQ-12 [34], personality traits, including Neuroticism, Agreeableness, Openness, Conscientiousness, and Extraversion, and diabetes status (0 for diabetes, 1 for healthy controls) as predictors of SRH. Interactions between personality traits and diabetes status were also included as predictors using the methodology outlined by Aiken and West [35]. Subsequently, two separate multiple regressions were conducted to understand the strength and directions of the associations between personality traits and SRH in diabetes patients and healthy controls, respectively: one with demographic variables, psychological distress, and personality traits as predictors, and SRH as the outcome variable for diabetes patients, and another with the same predictors and outcome variable for healthy controls.

## 3. Results

**Figure 1:**
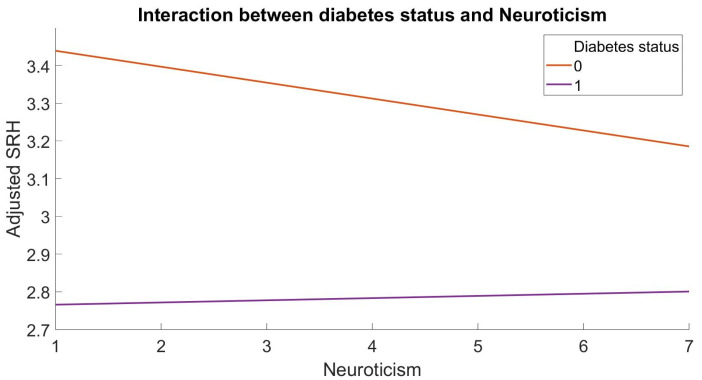
The interaction between diabetes status and Neuroticism in predicting self-rated health (SRH).

The present study observed a significant moderation effect of diabetes on the relationship between Neuroticism and SRH (b = 0.05, *p* < 0.05, 95% C.I. [0.02, 0.08]), while controlling for demographic variables and psychological distress (Figure 1).

Specifically, the regression model accounted for 23.2 percent of the total variances in SRH for the healthy control group. Among healthy controls, Neuroticism (b = −0.04, *p* < 0.001, 95% C.I. [−0.05, −0.03]) and Extraversion (b = −0.01, *p* < 0.05, 95% C.I. [−0.03, −0.001]) were negatively associated with SRH, whereas Openness (b = 0.02, *p* < 0.001, 95% C.I. [0.01, 0.04]) and Conscientiousness (b = 0.08, *p* < 0.001, 95% C.I. [0.07, 0.10]) showed positive associations with SRH. In contrast, for diabetes patients, the regression model explained 23.6% of the total variances in SRH, with only Conscientiousness demonstrating a positive relationship (b = 0.08, *p* < 0.001, 95% C.I. [0.03, 0.12]). Please refer to Table 2 for detailed results.

## 4. Discussion

The objective of this study was to examine the factors influencing SRH from a dispositional perspective using the Big Five personality traits in individuals with diabetes compared to healthy controls. Through hierarchical and multiple regression analyses, it was determined that diabetes significantly moderated the association between Neuroticism and SRH. Specifically, Neuroticism and Extraversion exhibited negative correlations with SRH, while Openness demonstrated a positive correlation with SRH. However, among diabetes patients, only Conscientiousness displayed a positive correlation with SRH.

The negative relationship between Neuroticism and SRH aligns with previous studies [22,36,37,38]. This can be attributed to the fact that individuals with high Neuroticism tend to have poorer overall health, which is reflected in their lower SRH scores. Behavioral markers, such as biological dysfunctions [39] and reduced walking speed (e.g., [40,41]), further support the negative relationship between Neuroticism and health. Additionally, Neuroticism is associated with adverse health outcomes, including major depression, dementia, and chronic respiratory diseases [42,43]. Moreover, individuals with high Neuroticism may perceive the world more negatively, influencing their tendency to rate their health lower than its actual state [44]. Shared genetic factors between Neuroticism and SRH have also been identified, as indicated by Harris et al. [5], who found a negative association between polygenic scores for Neuroticism and lower SRH. However, this study did not find a significant association between Neuroticism and SRH in diabetes patients, possibly due to the reduction in SRH already caused by diabetes, rendering certain dispositional traits irrelevant to SRH. In addition, diabetes can lead to various health complications, such as cardiovascular problems, nerve damage, and reduced immune function. These physical health burdens may reduce the impact of Neuroticism on SRH.

Surprisingly, no association between Agreeableness and SRH was found among healthy controls, consistent with some (e.g., [37]) but not other previous studies (e.g., [22,45]. It is plausible that the association between Agreeableness and SRH may be moderated by age, as most previous studies have examined this relationship across different age groups. However, further investigation is required to explore this aspect.

On the other hand, a positive correlation was observed between Openness and SRH among healthy controls. Individuals with high Openness tend to possess diverse interests, an appreciation for beauty and arts, and a preference for novelty over routine, which may contribute to their better overall health. Recent research has associated Openness with increased physical activity [44], improved physical functioning [40,41], and lower inflammation rates [9], subsequently leading to better SRH. The negative relationship between Extraversion and SRH in healthy controls is consistent with some studies [37,38,46], but can be explained by the fact that while most individuals with high Extraversion experience positive health outcomes through positive emotionality and social support [47], some may engage in risky behaviors, such as substance use, due to their inclination for seeking social experiences [48,49]. However, the association between Openness and SRH did not appear to be significant in people with diabetes, which can be explained by smaller sample size in diabetes, in which small associations could not be detected.

As anticipated, Conscientiousness exhibited a positive correlation with SRH in both healthy controls and diabetes patients, providing further evidence of the health benefits associated with this trait [50,51,52]. Conscientiousness is positively linked to health-promoting behaviors like physical activity [44,53]. Conversely, it is negatively associated with health-detrimental activities, including smoking [54] and alcohol use [55]. Previous research has also indicated that Conscientiousness is associated with a lower risk of chronic diseases [56], such as obesity [57] and depressive symptoms [42], while being positively correlated with indicators of physical health, such as lung function, grip strength, and walking speed [58,59]. These factors collectively contribute to better SRH.

Despite the strengths of this study, such as its large sample size, inclusion of healthy controls, and careful control of sociodemographic factors, there are some limitations to consider. The type of diabetes in the patient sample was not specified, and personality traits may relate differently to SRH in individuals with Type 1 and Type 2 diabetes. Furthermore, the study design was cross-sectional, preventing causal conclusions. Lastly, the generalizability of the findings beyond the United Kingdom may be limited. Future research should replicate these findings in other countries and consider longitudinal designs to establish causality.

## 5. Conclusions

In summary, this study aimed to investigate the relationship between personality traits and SRH in individuals with diabetes compared to healthy controls. The findings suggest that Neuroticism, Openness, and Extraversion may play distinct roles in predicting SRH, with differences observed between the two groups. Healthcare professionals and clinicians working with individuals with diabetes should consider the influence of personality traits on SRH. Tailored interventions can be developed to improve SRH based on their specific personality profiles. For example, interventions targeting healthy individuals high in Neuroticism could focus on managing emotional instability and providing support for coping with negative emotions. Conversely, interventions for people with diabetes low in Conscientiousness may emphasize the importance of adherence to diabetes management and self-care behaviors. Understanding the associations between personality traits and SRH can help healthcare professionals identify individuals at higher risk of experiencing poorer SRH outcomes. This knowledge can inform personalized care plans and interventions to promote better overall well-being in individuals with diabetes.

## Figures and Tables

**Table 1:** Descriptive statistics for healthy control and diabetes patients.

	Healthy Controls		Diabetes Patients	
	Mean	S.D.	Mean	S.D.
Age	58.37	11.58	58.70	15.66
Monthly income	1572.23	1600.37	1327.18	1026.78
SRH	3.36	1.12	2.67	1.12
GHQ-12	10.82	5.28	11.81	5.92
Neuroticism	3.38	1.45	3.44	1.51
Agreeableness	5.67	1.03	5.66	1.08
Openness	4.50	1.34	4.31	1.44
Conscientiousness	5.60	1.09	5.31	1.22
Extraversion	4.52	1.35	4.52	1.38
	N	%	N	%
**Sex**				
Male	6666	51.61	874	51.32
Female	6249	48.39	829	48.68
**Highest educational qualification**				
Below college	9164	70.96	1311	76.98
College	3751	29.04	392	23.02
**Legal marital status**				
Single	4791	37.10	698	40.99
Married	8124	62.90	1005	59.01

**Table 2:** The multiple regression models were employed to estimate the coefficients (b) and 95% confident intervals for healthy controls and individuals diagnosed with diabetes, with predictors including demographics, psychological distress, and personality traits, and the predicted variable being self-rated health (SRH). All numerical values were rounded to two decimal places.

	Healthy Controls	Diabetes Patients
Age	−0.02 *** [−0.02, −0.01]	−0.02 *** [−0.02, −0.02]
Sex	0.17 *** [0.14, 0.21]	0.12 * [0.02, 0.22]
Income (monthly)	0.00 *** [0.00, 0.00]	0.00 ** [0.00, 0.00]
Education	0.27 *** [0.23, 0.31]	0.16 ** [0.05, 0.28]
Marital status	0.15 *** [0.12, 0.19]	0.07 [−0.03, 0.16]
Psychological distress	−0.08 *** [−0.08, −0.07]	−0.07 *** [−0.08, −0.06]
Neuroticism	−0.04 *** [−0.05, −0.03]	−0.01 [−0.05, 0.02]
Agreeableness	0.00 [−0.02, 0.02]	−0.03 [−0.08, 0.02]
Openness	0.02 *** [0.01, 0.04]	0.02 [−0.01, 0.06]
Conscientiousness	0.08 *** [0.07, 0.10]	0.08 *** [0.03, 0.12]
Extraversion	−0.01 * [−0.03, 0.00]	0.02 [−0.02, 0.05]
R^2^	0.232	0.236

* *p* < 0.05, ** *p* < 0.01, *** *p* < 0.001.

## Data Availability

Publicly available datasets were analyzed in this study. These data can be found here: https://www.understandingsociety.ac.uk (accessed on 11 November 2022).
